# Chemical Proteomics for Target Discovery of Head-to-Tail Cyclized Mini-Proteins

**DOI:** 10.3389/fchem.2017.00073

**Published:** 2017-10-11

**Authors:** Roland Hellinger, Kathrin Thell, Mina Vasileva, Taj Muhammad, Sunithi Gunasekera, Daniel Kümmel, Ulf Göransson, Christian W. Becker, Christian W. Gruber

**Affiliations:** ^1^Center for Physiology and Pharmacology, Medical University of Vienna, Vienna, Austria; ^2^Division of Pharmacognosy, Department of Medicinal Chemistry, Uppsala University, Uppsala, Sweden; ^3^School of Biology/Chemistry, University of Osnabrück, Osnabrück, Germany; ^4^Department of Chemistry, Institute of Biological Chemistry, University of Vienna, Vienna, Austria; ^5^School of Biomedical Sciences, Faculty of Medicine, University of Queensland, St. Lucia, QLD, Australia

**Keywords:** cyclotides, cyclic protein, chemical proteomics, peptide-protein interaction, photo-affinity labeling

## Abstract

Target deconvolution is one of the most challenging tasks in drug discovery, but a key step in drug development. In contrast to small molecules, there is a lack of validated and robust methodologies for target elucidation of peptides. In particular, it is difficult to apply these methods to cyclic and cysteine-stabilized peptides since they exhibit reduced amenability to chemical modification and affinity capture; however, such ribosomally synthesized and post-translationally modified peptide natural products are rich sources of promising drug candidates. For example, plant-derived circular peptides called cyclotides have recently attracted much attention due to their immunosuppressive effects and oral activity in the treatment of multiple sclerosis in mice, but their molecular target has hitherto not been reported. In this study, a chemical proteomics approach using photo-affinity crosslinking was developed to determine a target for the circular peptide [T20K]kalata B1. Using this prototypic nature-derived peptide enabled the identification of a possible functional modulation of 14-3-3 proteins. This biochemical interaction was validated via competition pull down assays as well as a cellular reporter assay indicating an effect on 14-3-3-dependent transcriptional activity. As proof of concept, the presented approach may be applicable for target elucidation of various cyclic peptides and mini-proteins, in particular cyclotides, which represent a promising class of molecules in drug discovery and development.

## Introduction: background and objectives

Nature-derived peptides constitute diverse compound libraries and occupy an immense chemical space of different structures, conformations, chiralities, surface charges, and molecular interaction moieties (Nicolaou, 2014). Peptide natural products have been widely used in phenotypic drug screening to provide valuable lead compounds and biochemical probes (Arnison et al., 2013; Mullard, 2015). The peptide development pipeline currently includes several hundreds of compounds in the pre-clinical field, >50 candidates in clinical trials, and over 60 peptide drugs on the market (Bellmann-Sickert and Beck-Sickinger, 2010; Kaspar and Reichert, 2013; Harvey et al., 2015). One particular class of ribosomally synthesized and post-translationally modified peptides (RiPPs) are considered as future peptide drug candidates. Indeed, many of these natural products, or their nature-derived analogs, are stabilized via disulfide bonds, cystine-knots, and/or cyclization. They have advantages over their linear, mainly flexible, counterparts in terms of structural stability, target affinity, and pharmacokinetic properties, resulting in optimized therapeutic effectivity (Craik et al., 2012, 2013; Otvos and Wade, 2014). RiPPs have evolved during evolution as modulators for molecular proteins and receptors. For example, circular cystine-rich peptides, called cyclotides, recently attracted attention as orally bioactive peptide drugs (Craik et al., 2012; Thell et al., 2014, 2016). Ethnopharmacology and bioactivity-guided fractionation led to the discovery of native immunomodulatory cyclotides from the plant *Oldenlandia affinis*. Subsequent proliferation studies on isolated human T-cells *in vitro* yielded the synthetic peptide drug candidate [T20K]kalata B1 ([T20K]kB1) (Grundemann et al., 2012, 2013). Therapeutic efficacy of [T20K]kB1 has been recently highlighted for the treatment of autoimmune diseases in a mouse model of multiple sclerosis (Thell et al., 2016). Similarly, the cystine-stabilized polypeptide chlorotoxin isolated from the venom of the death-stalker scorpion (*Leiurus quinquestriatus*) is a peptide-drug candidate utilized in cancer diagnosis and therapy (Ojeda et al., 2016). Although, chlorotoxin-derived peptides have been approved for clinical trials, a defined protein target is still missing. This situation is a common issue in drug discovery of promising natural products, but clearly a risk and a bottleneck for further drug development (Dardevet et al., 2015). In fact, a key step in drug development is the identification of molecular target(s). Lack of knowledge about the molecular binding site(s) of drug candidates, restricts rational drug design and it ultimately reduces their attractiveness as chemical probes or pharmaceutical agents (Bottcher et al., 2010; Rask-Andersen et al., 2011). Photo-affinity labeling is one of the working horses in chemical proteomics for target identification (Gridling et al., 2014; Klaeger et al., 2016). The chemical tool box includes various linkers (e.g., polyethylenglycol), reactive sites (e.g., diazirines, benzophenones), and affinity labels (e.g., biotin, alkynes, and azides for click chemistry) for the design of suitable probes (Terstappen et al., 2007; Ziegler et al., 2013; Arrowsmith et al., 2015; Bunnage et al., 2015; Rylova et al., 2015). Despite, there are sophisticated methods and tools available for target deconvolution of small molecules and linear peptides including label-free as well as various labeling strategies for untargeted proteomics (Terstappen et al., 2007; Sletten and Bertozzi, 2009; Park et al., 2012; Ziegler et al., 2013), the decreased amenability of circular and stabilized peptides for chemical modifications, hinders target identification for these otherwise promising drug-like molecules. In fact, chemical modification, target capture, and affinity purification of highly structured circular peptides are not trivial. Therefore, we aimed to develop a robust workflow combining the design of cyclic and stabilized peptide probes with applications in target capture and crosslinking for the identification of interactions with cellular proteins.

In this article, we present a methodology for target deconvolution of circular and cystine-stabilized peptides using the prototypic immunomodulatory cyclotide kalata B1. We first designed a probe for photo-affinity labeling and chemical proteomics analysis to identify binding proteins of the cyclotide. After validation of the probe and the experimental protocol, we investigated protein interaction partners of kB1 in an immune proteome. As a proof of concept, we identified and confirmed the interaction of the cyclotide [T20K]kB1 and 14-3-3 proteins at biochemical and cellular level using co-precipitation experiments and a cellular reporter gene assay. This methodology is in particular applicable to other cyclotides, which constitute one of the most abundant and diverse class of nature-derived peptides with an estimated number of >150,000 peptides (Gruber et al., 2008; Hellinger et al., 2015b).

## Methodology: detailed experimental protocol

### Materials

Boc-L-(Ala; Arg-N_ω_-tosyl; Asn-*N*_γ_-xanthyl; Cys-S-4-methylbenzyl; Glu-O-5-benzylester; Gly; His-*N*_*im*_-tosyl; Ile, Leu; Lys-*N*_ε_-(2-chlorobenzyloxycarbonyl); Met; Pro; Ser-O-benzyl; Thr-O-benzyl; Trp and Val)-OH were from Iris Biotech (Marktredwitz, Germany); and Boc-L-Phe-benzoyl-OH was from Bachem (Bubendorf, Switzerland). Acrylamid/bisacrylamid solution 30/2.6% (w/w/v) was from Serva (Heidelberg, Germany). β-Mercaptoethanol, dimethylformamide, dimethylsulfoxid (anhydrous), formic acid (LC-MS grade) diisopropylethylamine, ammonium hydrogencarbonate, ammonium peroxodisulfate, *N, N, N*′*, N*′-tetramethylethylenediamine, bovine trypsin (sequencing grade), sodium hydrogencarbonate, disodium carbonate, trypsin-EDTA solution, Tween-20, RPMI-1640, calcium dichloride, magnesium sulfate, ethylenediaminetetraacetic acid, penicillin, streptomycin, and piperidine were from Sigma Aldrich (St. Louis, United States). Dichloromethane, acetonitrile (HPLC and LC-MS grade), methanol (HPLC synthesis or gradient grade), water (LC-MS grade), and 2-propanol (LC-MS grade) were from ChemLab (Bensheim, Germany). Trifluoroacetic acid, tris-(hydroxymethyl)-aminomethane, glycine, sodium chloride, sodium dodecylsulfate, acetic acid reduced, and oxidized glutathione and were from Carl Roth (Karlsruhe, Germany). Dulbecco's modified eagle high glucose medium was from GE Healthcare LifeSciences (Chalfont St. Giles, Great Britain). (2-(1H-benzotriazol-1-yl)-1,1,3,3-tetramethyluronium-hexafluorophosphat) (HBTU) was from PepChem (Wellington, United States).

### Peptide synthesis

Cyclotides were synthesized via solid-phase peptide synthesis using an automated Boc protocol on an ABI synthesizer A433 (Applied Biosystems, CA, United States). Boc-L-Leu-PAM resin (0.6 mmol/g substitution value, 200–400 mesh; Bachem) was allowed to swell in dimethylformamide (DMF) for 30 min before the generation of a thioester linker according to reported protocols (Camarero and Muir, 2001). The first manual amino acid coupling was achieved using 1 equivalent (eq) amino acid, dissolved within 2 min in 3.6 mL 0.5 M HBTU (9 eq in DMF) and the reaction was mixed well for 1 min after the addition of 29 eq diisopropylethylamine before adding to the resin. The coupling was allowed to proceed for 10 min. The Boc protecting group was removed with 5 mL TFA for 1 min, followed by a DMF wash and a quantitative analysis of the free amine groups using the ninhydrin reaction (Sarin et al., 1981). For all arginine and threonine residues double couplings were used twice and an extra DCM wash was applied after each glutamine coupling. After the final peptide elongation, resins were dried under a steady nitrogen flow. To cleave the peptide off the resin, scavengers *p*-cresol and *p*-thiocresol were added in a ratio of 100:8:2 (v/w/w) relative to the applied liquid HF. The resins were cooled to −78°C using a dry ice-ethanol mixture to condense about 10 mL HF. Subsequently, the temperature was raised to 0°C and the cleavage was achieved within 1 h. The cleaved linear peptide was precipitated and washed with ice-cold diethyl ether and dissolved again in CH_3_CN/H_2_O/TFA 75/25/0.1% (v/v/v). Lyophilized crude peptide (0.1 mg/mL) was used for oxidative folding in a buffer containing 0.1 M NH_4_HCO_3_ at pH 8.2/iPrOH 1:1 (v/v), as well as reduced/oxidized glutathione (GSH/GSSG 2/0.5 mM). The folding process via chemical ligation is potentially supported by intramolecular thiolactone formation, previously described as thia-zip mechanism (Tam and Lu, 1998). The reaction was allowed to proceed for 24 h at 22°C.

### Peptide modification

Biotinylation of cyclotide probes was achieved with N-hydroxysuccinimide-LC-LC-biotin reagent from (Thermo Scientific). Peptide was dissolved in 0.1 M NaHCO_3_/Na_2_CO_3_ pH 8.8 and incubated for 8 h at 22°C with a 20-fold molar excess of reagent (prepared in anhydrous dimethylsulfoxid; Sigma Aldrich). The reaction was quenched with 0.1% (v/v) TFA.

### Peptide purification and analysis

A Dionex Ultimate 3000 HPLC was used for chromatographic reversed phase purifications (Thermo Scientific). Native folded cyclotide was purified by (semi-) preparative chromatography using a Dichrom Kromasil C_18_ column (250 × 20.2 mm, 10 μm), and a Dichrom Kromasil C_18_ column (250 × 10 mm, 5 μm) at 8 and 3 mL per min, respectively. Mobile phases were 0.1% aqueous TFA (solvent A) or CH_3_CN/ddH_2_O/TFA, 90/10/0.1% (v/v/v) (solvent B). Peptides were separated by linear gradients from 5 to 65% solvent B (1%/min) and monitored at A_214_, A_254_, and A_280_. Resulting fractions and peptides were lyophilized and analyzed by analytical HPLC using a Phenomenex Kinetex column (150 × 3 mm, 2.1 μm). Final peptide purity was >90% as judged by the A_280_-trace. Modified peptides were directly purified using semi-preparative HPLC as described above. The concentrations of peptide solutions were calculated using Beer-Lambert law at A_280_ (ε_[T20K]kB1_ = 6410 L M^−1^ cm^−1^). The extinction coefficients for the benzophenone incorporated probes were calculated based on amino acid analysis of a defined peptide solution.

### MALDI-TOF analysis of peptides

Peptide molecular weight analysis was performed with a MALDI-TOF MS 4800 analyzer from ABScieX (Framingham, MA, United States). Spectra were generated in the positive reflector mode. The mass spectrometer was calibrated using external calibration with Peptide Mix 1 from Laserbiolabs (Sophia-Antipolis, France) daily. Acidified samples were mixed with α-cyano-hydroxy-cinnamic acid matrix dissolved in CH_3_CN/ddH_2_O/TFA 50/50/0.1% (v/v/v) in a ratio of 1:6 and 0.5 μL of the mixture was spotted on the target plate.

### NMR structural characterization

Dried peptides (1 mg; ~1 mM) were dissolved in 600 μL of H_2_O/D_2_O (9:1, v/v) at pH ~5.2. One- and two-dimensional spectra (^1^H TOCSY with a mixing time of 80 ms and ^1^H NOESY with a mixing time of 200 ms) were obtained at 298 K and at 800 MHz. All data were recorded and processed using Topspin (Bruker). The water signal was suppressed using a modified WATERGATE sequence (Rosengren et al., 2003). Generally, 4,096 data points were collected in the F2 dimension and 256 (128 complex) points in F1, with 512 increments of 8 scans over 11,194 Hz. NMR spectra of [T20K]kB1 and [T20K,W23Bpa]kB1 were recorded and chemical shifts were determined by sequential assignment strategies. Initially, individual spin systems were identified in the NH-H^α^ fingerprint region of the TOCSY spectrum. The sequential resonance assignments were achieved by linking individual spin systems via sequential d_αN(i,i+1)_ connectivities in the fingerprint region of the NOESY spectrum. The secondary structures of the peptides were predicted using secondary H^α^ chemical shifts. For H^α^-chemical shift comparison of the peptides measured H^α^-values were corrected with the Δ (experimental water signal/expected water signal, 4.755) and the differential values to a random coil conformation were plotted (Wishart et al., 1995). For comparison kB1-values were taken from the literature and medium and long range NOEs were used to support the predicted secondary structure elements (Merutka et al., 1995; Saether et al., 1995; Rosengren et al., 2003; Barry et al., 2004).

### Cell culture and transfection

Jurkat cells were cultured in RPMI medium containing phenol red supplemented with 10% (v/v) heat deactivated fetal calf serum and 100 U/mL penicillin/streptomycin at 37°C in humidified atmosphere of 5% CO_2_. HEK293 cells were cultured in Dulbecco's modified eagle high glucose medium supplied with 10% fetal calf serum and 100 U per mL penicillin/streptomycin. Cell transfection was carried out by Jetprime® according to the manufacturer's recommendation (Polyplus, Illkirch, France).

### Bioactivity confirmation of chemical probes

Biological integrity of probes was determined using their reported immunosuppressive activity (Grundemann et al., 2013; Thell et al., 2016). Briefly, activity was measured in isolated lymphocytes derived from self-donated human venous blood or mouse splenocytes derived from 2D2 myelin oligodendrocyte glycoprotein specific T-cell receptor transgenic mice on a C57Bl/6 background [C57Bl/6-Tg(Tcra2D2,Tcrb2D2) Kuch/J]. These mice develop spontaneous experimental autoimmune encephalomyelitis, a mouse model of multiple sclerosis. Bioactivity was assessed by T-cell proliferation using FACS or cytokine concentration analysis of interleukin-2 in the supernatant of *ex vivo* re-stimulated splenocytes for 72 h using an ELISA kit and antibodies from eBioscience® (Grundemann et al., 2013; Thell et al., 2016). All experiments were approved according to the European Community rules of animal care with the permission of the Austrian Ministry of Science (BMWF-66.009/0241-II/3B/2011).

### Protein extraction and analysis

Splenocytes or Jurkat cells were lysed with 25 mM Tris-HCl pH 7.4, 40 mM KCl, 2.5 mM MgSO_4_ 2.5 mM CaCl_2_, 2% (v/v) glycerol, 1 mM EDTA under repeated freeze-thaw cycles and 10 min sonification. All experiments were performed in buffer with 1x complete protease inhibitor cocktail (Hoffman-La Roche, Basel, Switzerland). Cell membranes and debris were pelleted for 30 min at 16,000 × g and the supernatant corresponds to the “soluble cell lysate.” Protein concentration was determined using a bicinchoninic acid assay kit (Thermo Scientific) and DTT (1 mM) was added to keep redox potential constant during binding experiments. Photo-affinity pull down samples were submitted to denaturing sodium dodecyl sulfate polyacrylamide gel electrophoresis (PAGE). For immunoblotting according to earlier published protocols (Gruber et al., 2007; Bergmayr et al., 2013) proteins were semi-dry transferred onto a nitrocellulose membrane 0.45 μM (Amersham Protran, GE Healthcare, Austria) using a Fast Semi-dry blotter (Bio-Rad) at 0.25 mA per gel for 1 h. Primary rabbit anti-GFP antibody (1:5,000) was from Invitrogen (A6455), rabbit anti-GST antibody (1:2,500) from Abcam (ab9085), and mouse anti-human calcineurin subunit A antibody (1:2,000) clone BE2.1 from eBioscience. Secondary ECL anti-mouse (NA931V) or ECL anti-rabbit (NA934VS) IgG antibodies (1:10,000) were conjugated with horse radish peroxidase (GE Healthcare). Chemo-luminescence signals were detected on an imaging system FluorChem HD2 (Alpha Innotech, CA, United States). For silver staining protein gels were treated immediately after electrophoresis with fixing solution EtOH/ddH_2_O/acetic acid 50/40/10% (v/v/v) for 20 min. Gels had to be washed acid free, first with 50% (v/v) EtOH for 10 min and with ddH_2_O for 2 h. Gels were exposed to sensitization buffer 0.025% Na_2_S_2_O_3_ in ddH_2_O for 3 min, followed by 2 min wash with ddH_2_O. Gels were silver loaded using a 0.15% AgNO_3_ solution for 45 min in the dark. The protein bands were stained in development buffer formaldehyde/Na_2_CO_3_ 0.04/2% (v/w) under rigorous shaking. The reaction was quenched with 5% (v/v) acetic acid solution and the gels were stored until further processing in the stopping buffer at 4°C. Pixel quantification was carried out using ImageJ v1.47 (National Institute for Health).

### Pull down experiments

Biotin-LC-LC-labeled (ThermoFisher) peptides (1–5 nmoles) with or without (non-biotinylated) competitor (20–100-fold molar excess) were incubated with Jurkat cell or murine splenocyte cell lysates (150–500 μg proteins) for 2 h at 4°C in the presence of protease inhibitors. UV-activation of photo-crosslinking moiety benzophenone was induced by irradiation at 360 nm on ice for 15 min (Stratalinker, Stratagene, CA, United States). Streptavidin-coated magnetic nanobeads 2–4 mg (1–3-fold excess of binding capacity to applied biotin-bait) facilitated the precipitation (Hyglos, Bernried, Germany). The binding could proceed overnight at 4°C. The supernatants were removed and the beads were washed several times, unless stated otherwise and used for SDS-PAGE, unless stated otherwise. For cyclotide experiments the following peptides were used: biotinylated [T20K]kB1 or [T20K,W23Bpa]kB1 as bait and [T20K]kB1, [T20K,W23Bpa]kB1, or R18 (PHCVPRDLSWLDLEANMCLP; Sigma Aldrich), a 14-3-3 family specific peptide inhibitor, as competitors. Generally, beads were washed with lysis buffer containing 1% (v/v) Triton-X100 and 0.2% SDS (w/v) and protease inhibitor cocktail for 3 h followed by wash with 0.12 mM phosphate buffered high saline (PBhS) pH 7.4, 1 M NaCl and 12 mM KCl, 1 mM EDTA for 2 h. Lastly, the beads were washed three times for 5 min with lysis buffer. After photo-crosslinking the beads were washed with lysis buffer containing 2% (v/v) Triton-X100 and 0.2% SDS (w/v) and protease inhibitor cocktail for 3 h followed by wash with 0.12 mM PBhS pH 7.4, 2 M NaCl, and 12 mM KCl, 1 mM EDTA, 10% (v/v) glycerol for 2 h. Photo-crosslinking was also performed in Jurkat cells (2 × 10^6^) that were cultured in complete RPMI medium at 37°C in humidified atmosphere with 5% CO_2._ Bait peptide [T20K,W23Bpa]kB1 with or without 60-fold molar excess of competitor [T20K]kB1 were added to the suspension for 2 h. The cells were re-suspended in serum free RPMI medium and irradiated for probe conjugation to proteins. Cells were harvested by pelleting via centrifugation for 5 min at 300 × g. Peptide-protein co-precipitations of 14-3-3 proteins and cyclotides were analyzed with GST-tagged 14-3-3β protein (0.25 μg; EnzoLifeSciences, New York, United States). They were incubated with biotinylated bait peptides (10-fold excess of binding capacity) with or without the presence of competitors (150–500-fold molar excess) [T20K]kB1, [T20K,W23Bpa]kB1, or R18 in lysis buffer for 2 h at 4°C. The beads were washed with (i) lysis buffer only, (ii) lysis buffer containing 3% (v/v) Triton-X100 and 0.4% SDS (w/v) and protease inhibitor cocktail, (iii) 0.12 mM PBhS pH 7.4, 2 M NaCl, and 12 mM KCl, 1 mM EDTA, 10% (v/v) glycerol, (iv) 6 M guanidine hydrochloride in PBS, (v) 2% (w/v) SDS in PBS, (vi) 4 M urea and 2 M thiourea, 1% (w/v) SDS in PBS, and (vii) 0.1 M Tris-HCl, 140 mM NaCl, pH 10. The beads were eluted and applied to denaturing gel electrophoresis and immune blotting using an anti-GST antibody and streptavidin-conjugated horse radish peroxidase (dilution 1:4,000).

### Sample preparation for mass spectrometry

Single protein bands of a silver-stained gel (soaked in 5% acetic acid) were in-gel digested. The bands of interest were destained with 15 mM K_3_Fe(CN)_6_ and 50 mM Na_2_S_2_O_3_ in ddH_2_O for 5 min under agitation and washed four times (5–10 min) with ethanol/ddH_2_O/acetic acid 50/40/10% (v/v/v). Colorless gel pieces were dissolved in digestion buffer 50 mM NH_4_CO_3_ buffer for 5 min under agitation. Thiol reduction was performed with 10–20 mM DTT for 30 min at 56°C, followed by carbamidomethylation with 50–75 mM iodoacetamide in digestion buffer for 20 min at 23°C in the dark. Gel pieces were dehydrated using CH_3_CN and remaining solvent was evaporated using a SpeedVac (Savant Speedvac AES1010). Gel pieces were rehydrated on ice with trypsin solution (12.5 ng/μL; in digestion buffer) and enzymatic digest was allowed to proceed at 37°C without shaking overnight. Peptide fragment extraction was induced by adding 20 μL digestion buffer and a 15 min sonication treatment at 4°C. The supernatant was collected in a fresh vial and 20 μL of extraction buffer CH_3_CN/ddH_2_O/formic acid 49.75/49.75/0.5% (v/v/v) were added twice to the remaining gel pieces. Combined extracts were concentrated to reduce the total volume to about 20 μL. Digested samples were kept at −20°C before analysis. For in-solution digest, the beads were equilibrated with digestion buffer, followed by reduction/carbamidomethylation, and protein digestions using trypsin.

### LC-MS analysis

For proteomics application and protein identification trypsinized peptides were chromatographed on a Dionex Ultimate 3000 RSLC HPLC fully equipped to run microliter flowrates. Separation was achieved on PepMap Acclaim RSLC (250 mm × 75 μm, 2 μm; both from Thermo Scientific). The system is equipped with a pre-concentration and desalting cartridge where 0.1% TFA is used as transport liquid. Separation used flow rates of 500 nL per min with solvent A 0.1% aqueous formic acid and solvent B CH_3_CN/ddH_2_O/formic acid 80/20/0.08% (v/v/v). Linear gradients from 4 to 55% solvent B within 120 min were applied for peptide separation.

### Tandem MS experiments

Bottom-up shotgun proteomics was performed on a QqTOF mass spectrometer oTOF compact from Bruker Daltonics (Billerica, MA, United States) using the oTOF control software v3.4 (build16). Nano-flow captive spray device served as interface for sample ionization. The device was externally calibrated in the enhanced quadratic mode using the low concentration calibration mix (Agilent Technologies) in a range of 118 to 2,200 Da before starting sequential analysis. Additionally, high mass accuracy was achieved by an internal calibration based on the lock mass calibration with the calibrant hexakis-(1H,-1H,-4H-hexafluorobutyloxy)-phosphazine (Agilent Technologies). Detector resolution, ion transfer and detector gain were tuned semi-automatic on a weekly basis using the calibration mix. Source and ion transfer parameter were optimized to give maximal signal over the whole mass signal range. The cycle time for MS1 and MS/MS fragmentation was 3 s and exclusion criteria were maintained to reduce triggering of background signals. Scan range for precursor recording was set to 50−2,200 m/z with a minimal precursor mass of 200 m/z. Peptides carrying positive charges from 2 to 5 were set to trigger MS/MS events. An absolute threshold of 3,000 counts was used for signals to be considered for a MS/MS event. High energy collision induced dissociation was performed under the regime of a set energy set. These pre-setting uses optimized collision energy for precursor signals based on the parameters: isolation m/z, isolation mass range width, and charge state of the ion.

### Protein database searching

Peak list generation was conducted by the Compass Data Analysis software v4.2 (Build 395) (Bruker Daltonics). The peptide assignment to proteins were conducted with the Proteinscape software v.3.1.5 474 (Build 0140711-1459) (Bruker Daltonics) which makes use of the Mascot algorithm version 2.5 (Matrixscience, MA). Peptide fragmentation search were against human or mouse UniProtKB/Swiss-Prot protein database (last update 07/2016, number of protein entries 551705; http://www.ebi.ac.uk/uniprot). Parameters for the peak list generation were: enzyme specificity: trypsin, species: human, peptide tolerance ±10 ppm, MS/MS tolerance ±0.05 Da, number of considered ^13^C atoms 1, charge states 2^+^–5^+^, up to one missed cleavage, fixed modifications carbamidomethylation on cysteines, variable modifications deamidation of glutamine and asparagine residues and oxidation of methionine. In case of MS/MS spectra matching to peptides of more than one protein, the rank 1 peptide was considered for the assignment. Peptide ion score cut-off for peptide Mascot assignments to proteins were set to 15 and variable modifications were added as search option, i.e., phosphorylation (STY) and N-acetyl (K, N-terminal). The probability score applied of the search algorithm was set to *P* < 0.05. For protein validation of in-gel digested samples exclusion criteria supported efficient target identification by considering the following: only proteins identified in at least two independent analyses were considered as possible binding protein in the test pull down experiments. Furthermore, typical background or impurities proteins (i.e., keratins) were excluded as well as proteins identified with an unexpected difference to the assumed molecular mass of the gel piece (possible protein fragments). The MS proteomics data have been deposited at the ProteomeXchange Consortium via the PRIDE partner repository with the data set identifier PXD004798 (project DOI: 10.6019/PXD004798; Vizcaino et al., 2013).

### Reporter gene assay

Forkhead box protein 3a induced transcriptional activity was measured on transiently transfected HEK293 cells (5 × 10^4^). The reporter plasmid encoding luciferase under the control of a promotor containing forkhead responsive elements and the human p-Foxo3a-HA plasmid were gifts of Michael Greenberg provided by the Addgene consortium (#1787 and #1789; Brunet et al., 1999). The plasmids encoding the mouse p-14-3-3 beta-GST protein and the functional forkhead transcription factor were used in a 1.5-fold amount compared to the reporter construct. The transfection was completed after 6 h and the cells could recover for 4 h in growth medium. The cells were serum starved using 1% (v/v) fetal bovine serum in phenol red free DMEM medium supplemented with antibiotics overnight. After 4 h peptide and 24 h of fusicoccin-A treatment the luciferase assay was developed using luciferase development kit from Promega E151 (Wisconsin, United States) and the luminescence was measured on a synergy H4 plate reader (Biotek). Control compounds were the PI3K inhibitors wortmannin and LY294002 (both Sigma Aldrich). The recorded data were normalized to the non-treated control sample. Significance and IC_50_-values were calculated from at least two biological replicates using GraphPad Prism. Data analysis was performed by non-linear regression using a four parameter Hill equation.

### Data analysis and statistics

If not stated in detail for the respective single experiment the statistical tests were performed using GraphPad Prism 5.04 (GraphPad, La Jolla, CA) using one-way ANOVA with Bonferonni or Dunnett's *Post-hoc* test. The number of technical and biological repeats was justified by analysis of error and reproducibility of results. Significance threshold *P* < 0.05 was applied to all experiments. For the data shown in **Figure 3** three individuals for the *in vivo* experiments were used and each data point was performed in three technical replicates. The pull down experiments shown in **Figures 4A,B** were performed a single time and the data represent two technical repeats. Gel lanes were loaded with single (1x) or doubled (2x) amount of pull down proteins for better visibility. The data illustrated in **Figures 4C,D** were performed in two independent experiments. Silver stained gel lanes were evaluated by loading various amounts, and then the amount with optimized staining contrast was loaded and used for in-gel protein digestion. The pull down experiments shown in **Figures 4E,F** were performed a single time. The Western Blots and the pull down experiment shown in **Figure 5** represent experiments in three and two independent experiments, respectively. Experiments in a well-based format (**Figure 6**) were performed with three technical and two biological replicates. All data illustrated in this manuscript show the mean ± standard deviation (SD) unless stated otherwise. The MS based protein identifications were performed in two technical analyses for each sample.

## Results: tool design and validation of the method

### Synthesis of peptide probe

**Figure 1:**
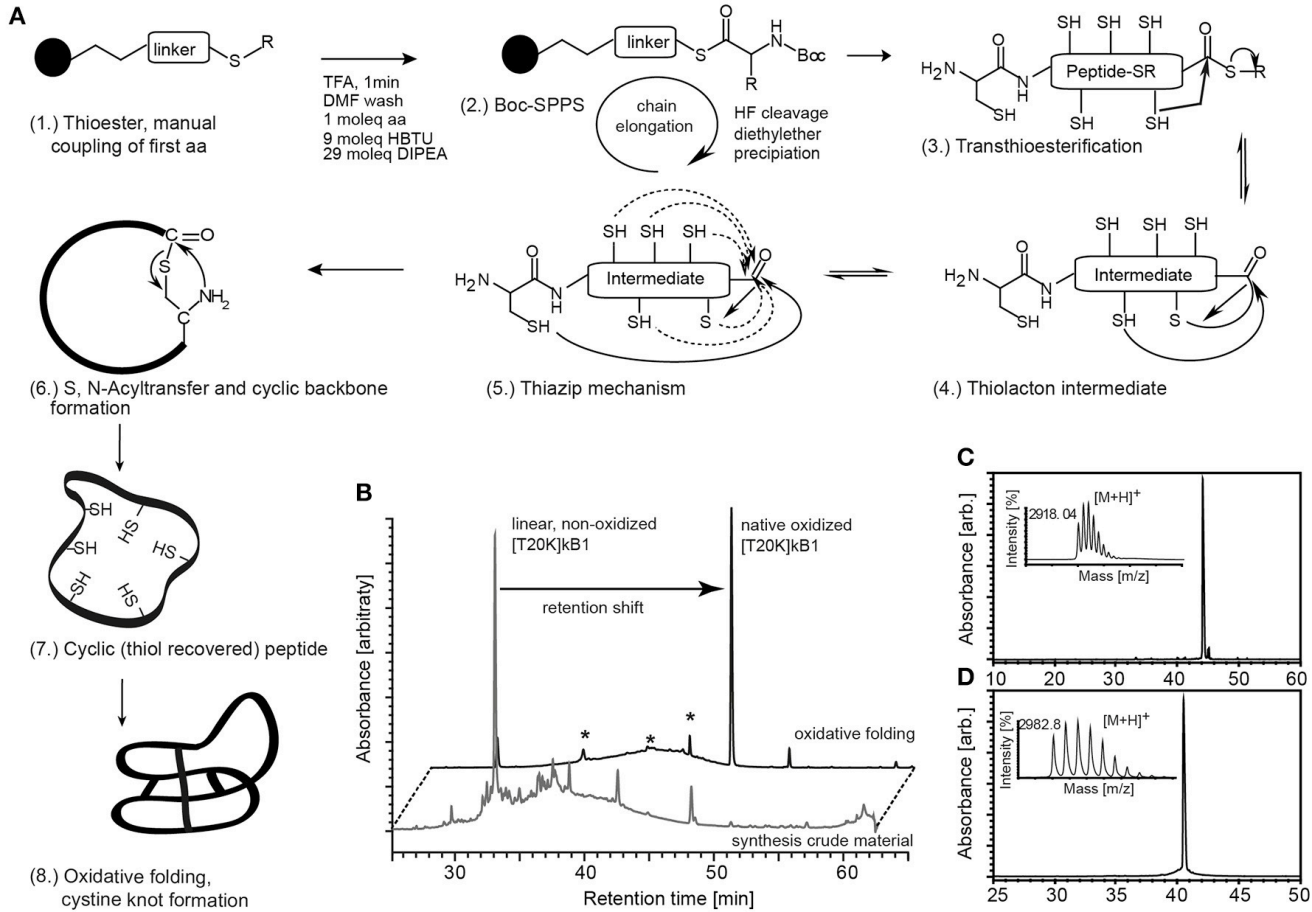
Synthesis of circular peptide probes. **(A)** (1.) Cyclotides and probes were synthesized as linear thioester precursor peptides using a Boc-solid-phase peptide synthesis protocol. (2.) Peptide precursor was released from the resin and globally de-protected by hydrogen fluoride (HF) treatment. The cyclic native folded peptide was obtained after native chemical ligation potentially supported via the thia-zip mechanism described for cysteine-rich peptides in oxidative folding conditions. (3., 4.) Transthioesterification forms an intramolecular thiolactone intermediate. (5.) The proposed thia-zip mechanism brings the N-terminal cysteine residue in close proximity to the C-terminal thiolactone intermediate. (6., 7.) An irreversible S,N-acylshift forms a native peptide bond and recovers all thiols. (8.) The native cystine-knot fold is achieved in oxidative folding buffers. **(B)** Native folded cyclotides exhibit a shift in chromatographic retention compared to its reduced progenitor and all identified non-native folded species (marked with asterisks) as indicated by the A_280_ UV traces of the peptide before and after oxidative folding. Synthesized and oxidative folded cyclotide probes [T20K]kB1 **(C)** and [T20K,W23Bpa]kB1 **(D)** were purified via preparative HPLC and evaluated for their molecular weight using MALDI-TOF mass spectrometry and for purity by analytical HPLC analysis. Mass spectra and UV chromatograms (A_280_) are presented. Retention time and molecular weight (monoisotopic [M+H]^+^) are indicated (small inserts).

To identify a molecular target of kalata-cyclotides, a chemical probe incorporating a Lys residue for labeling and an UV active crosslinker moiety as artificial amino acid side chain were generated using Boc-solid-phase peptide synthesis for the synthesis of circular cystine-rich peptides. Based on previously established structure-activity data of the immunomodulatory cyclotide kB1 (Grundemann et al., 2013) we selected rational sites Thr^20^ for lysine modification and biotinylation (T20K) and Trp^23^ for Bpa modification and crosslinker site, respectively. Cyclotide [T20K]kB1 and photo-reactive crosslinker probe [T20K,W23Bpa]kB1 (Table 1) were prepared applying a protocol for the synthesis of thioester precursor peptides. Native circular cystine-knot fold of the cyclotide was achieved after oxidative folding of the fully de-protected thioester precursor by native chemical ligation (Figure 1).

**Table 1:** Cyclotide probes.

**Name**	**Sequence^a^**	**Molecular weight (Da) (mono.)**
kalata B1 (kB1)	CGETCVGGTCNTPGCTCSWPVCTRNGLPV	2890.14
[T20K]kB1	CGETCVGGTCNTPGC**K**CSWPVCTRNGLPV	2917.19
[T20K, W23Bpa]kB1	CGETCVGGTCNTPGC**K**CS**X**PVCTRNGLPV	2981.86

a *X indicates the amino acid replacement with p-benzophenylalanine (Bpa)*.

### Structural characterization of the functionalized probe

**Figure 2:**
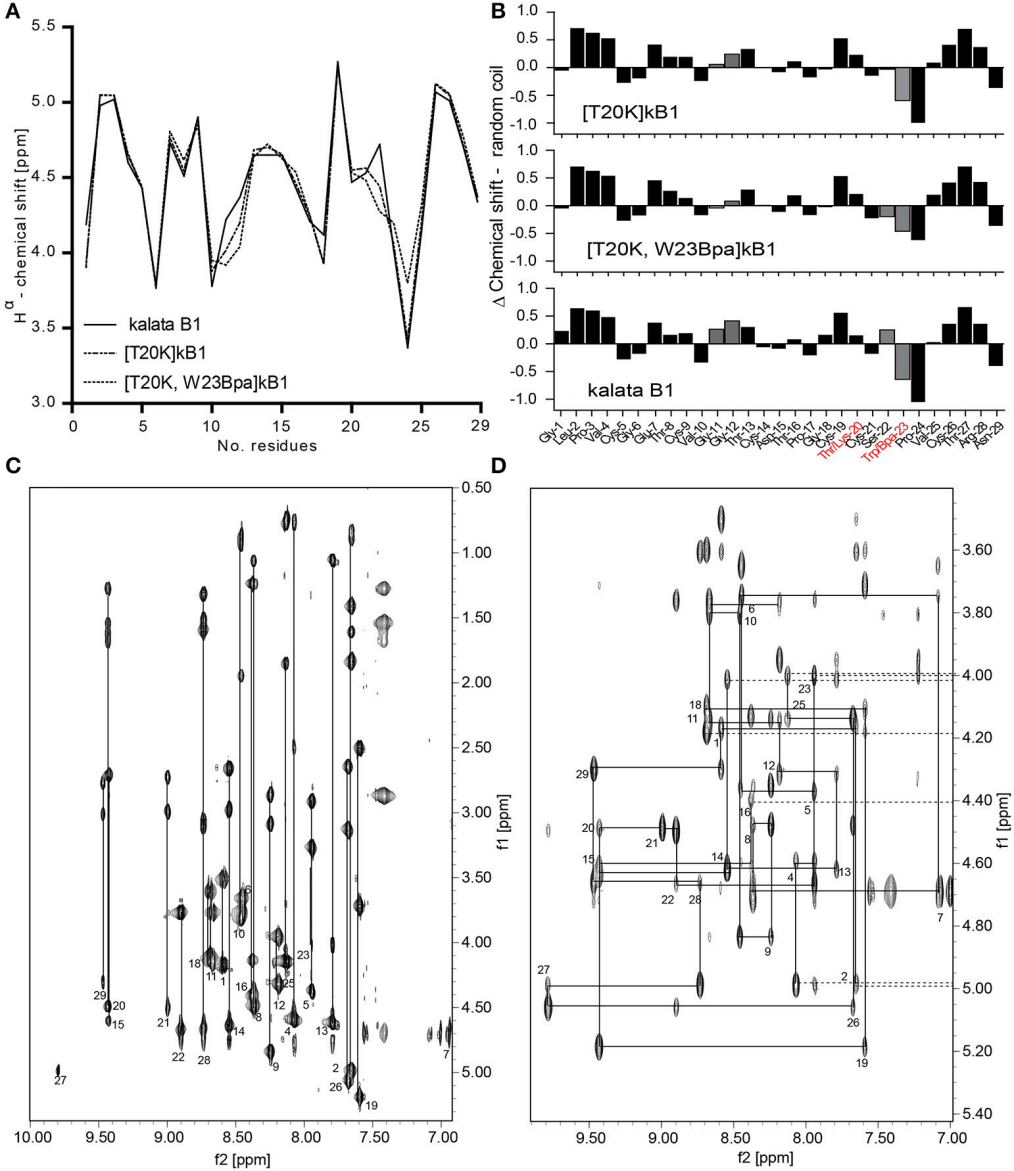
Structural integrity of the circular peptide probe. **(A)** Superimposed H^α^-chemical shifts of kalata B1 (kB1), [T20K]kB1, and [T20K,W23Bpa]kB1 are shown. **(B)** Secondary structure elucidation of the cyclotide probe [T20K,W23Bpa]kB1 in comparison to endogenous kB1 and the reference mutant [T20K]kB1 as determined by examining H^α^-proton shifts relative to the random coil configuration. Delta (Δ)-values (random coil—experimental values) are presented where numerical values ± 0.1 ppm suggest a well-defined structure. Mutations sites are highlighted in red characters. The recognized H-proton shifts for residues Gly^11^-Gly^12^ and Ser^22^-Trp^23^ are indicated with gray filled bars. **(C)** NH-H^α^ fingerprint region of the TOCSY spectrum of [T20K]kB1 showing intraresidue connectivities. **(D)** Sequential d_αN(i,i+1)_ connectivities in the NH-H^α^ fingerprint region of the NOESY spectrum for [T20K]kB1.

A crosslinker and an affinity tag were incorporated into the peptide [T20K]kB1. To evaluate the structural and conformational integrity of the designed probes, structural studies of [T20K]kB1 and the representative cyclotide-like probe [T20K,W23Bpa]kB1 were performed based on secondary H^α^-shifts derived from NMR spectroscopy (Figure 2A). The original report untying the structure of kB1 served as reference for comparison since the investigated peptides [T20K]kB1 and benzophenone-derivatives are based on the same core structure (Saether et al., 1995). H^α^-chemical shifts were examined for cyclotide specific structural features: cyclic backbone, cystine-knot, anti-parallel β-strands, β-hairpins and the *cis*-Pro backbone twist. The spectra showed well-dispersed resonances, confirming that both synthetic peptides, [T20K]kB1 and [T20K,W23Bpa]kB1 were correctly folded and highly structured in solution (Figure 2B).

The cyclic nature of both peptides was unequivocally confirmed by a continuous closed series of sequential NOE connectivities, including d_αN_, d_NN_, d_αδ_ (*trans*), or d_αα_ (*cis*) in case of prolines. Figures 2C,D show the intraresidue region of the TOCSY spectrum and finger print region of the NOESY spectrum for [T20K]kB1 to illustrate exemplarily the procedure of sequence assignments. The cyclotide fold is described as an elongated peptide circle that folds back on top of itself and is stabilized by three cross-bracing disulfide bonds, which link diagonally opposed β-strands (Rosengren et al., 2003). The main element of secondary structure in cyclotides is an antiparallel β-sheet. Analysis of the secondary H^α^ chemical shifts suggested that both [T20K]kB1 and [T20K,W23Bpa]kB1 contain secondary structure elements, namely, two antiparallel β-strands. Strand 1 (Gly^18^-Ser^22^) and strand 2 (Val^25^-Arg^28^) appear connected by a turn (Ser^22^-Val^25^) forming a traditional β-hairpin motif as described for kB1 (Rosengren et al., 2003). Supporting the presence of a β hairpin motif, several long range strong NOEs, d_αN(20, 27)_, d_NN(20, 27)_, d_NN(22, 27)_, d_αN(22, 27)_ in [T20K]kB1 NOESY spectra could be identified. Although the above NOEs could not be unequivocally identified in [T20K,W23Bpa]kB1 spectra, the presence of strong inter-strand long range NOEs, d_αN(19, 27)_ and d_αN(22, 28)_ were supportive for the presence of a β hairpin motif. Both sequences also incorporate three proline residues. Strong sequential H^α^-H^δ^ NOEs cross-peaks for Leu^2^-Pro^3^ and Thr^16^-Pro^17^ confirmed *trans*-peptide bonds, where as a strong sequential H^α^-H^α^ cross peak between Trp^23^/Bpa^23^ and Pro^24^ confirmed a *cis*-peptide bond consistent with what has been previously reported for Möbius type peptides with the presence of *cis*-backbone twist.

Both [T20K]kB1 and [T20K,W23Bpa]kB1 have almost identical secondary H^α^-chemical shifts throughout the sequence except for minor deviations; in comparison to [T20K]kB1, a significant deviation in secondary H^α^ is observed at residue 23 (loop 5) where the Trp to Bpa mutation is introduced. In addition slight deviation in chemical shift is observed for Gly^12^ (loop 2), indicating the mutation introduced in loop 5 has an influence on Gly^12^ in loop 2. It is of note that loops 2 and 5 residues are known to be clustered together in two β-turns immediately adjacent to each other (Craik et al., 1999), thus it is quite likely that residues in these two loops participate in local interactions. The presence of the Bpa side chain was clearly evident by strong TOCSY peaks, d_αN(23, 23)_ and d_βN(23, 23)_ in the TOCSY spectra as well as d_βδ(23, 23)_ in the NOESY spectra, respectively.

### Bioactivity of the functionalized probes

**Figure 3:**
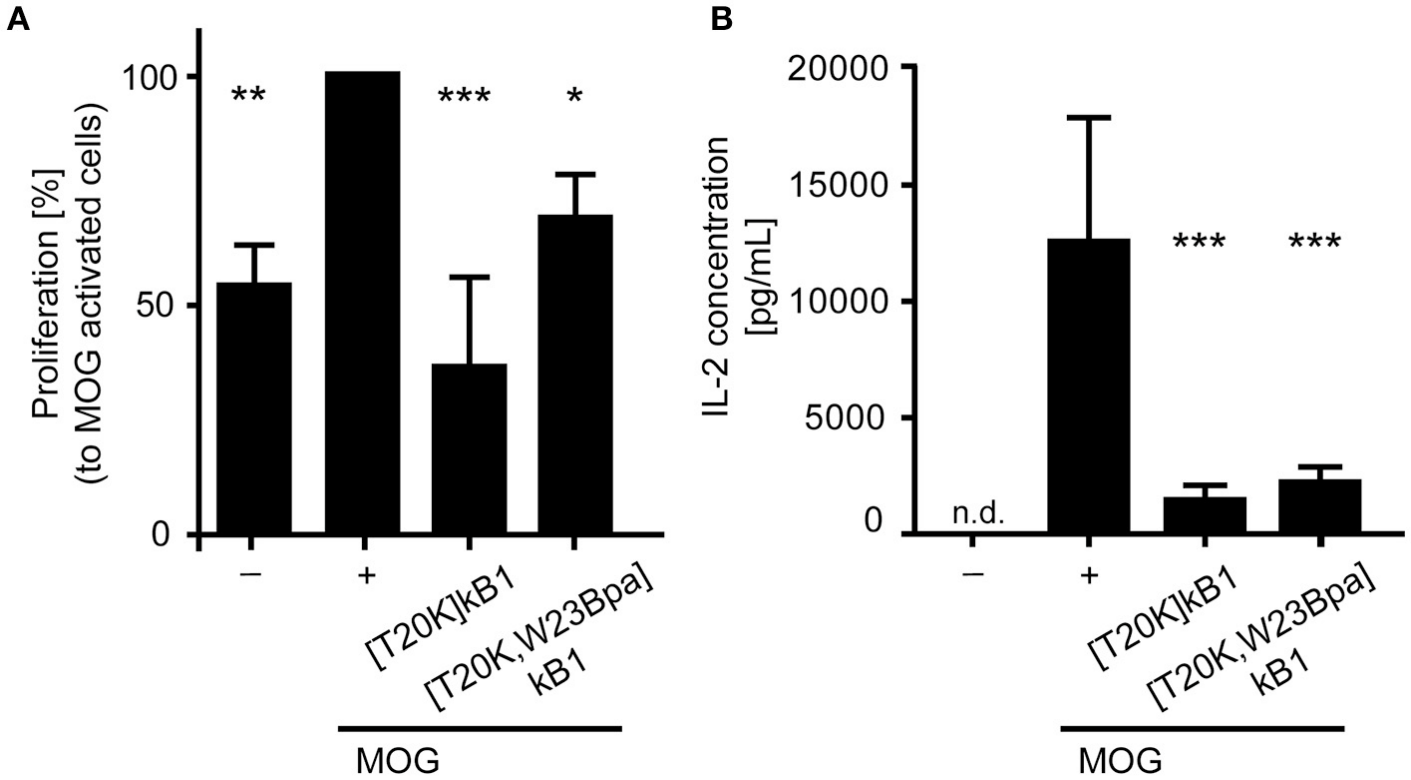
Biological activity of the circular peptide probe. Bioactivity was assayed by **(A)** anti-proliferation assays of myelin oligodendrocyte glycoprotein (MOG)-immunized mouse splenocytes and **(B)** extracellular IL-2 cytokine levels of MOG-activated splenic cells from 2D2 mice. The cyclotide probe [T20K,W23Bpa]kalata B1 (kB1) was measured in comparison to [T20K]kB1 (4 μM each), a MOG-stimulated positive control (+) and an unstimulated cell only control (−). The proliferation data are normalized relative to the MOG-stimulated control. Data are shown as mean ± S.D. of three independent biological experiments. Significance was evaluated using one-way ANOVA with Bonferonni or Dunnett's *Post-Hoc* test (^*^P < 0.05, ^**^P < 0.01 and ^***^P < 0.001); n.d., not detected.

The probes were further assessed for their bioactivity *in vitro* to reduce proliferation on myelin oligoglycoprotein restimulated splenocytes isolated from 2D2 diseased mice. Assayed cyclotide probes induced a suppressive effect on proliferation of splenocytes with 36.7 ± 19.4% ([T20K]kB1) and 69.2 ± 6.7% ([T20K,W23Bpa]kB1) as compared to the myelin oligoglycoprotein activated positive control (Figure 3A). Moreover, examination of the interleukin-2 cytokine levels upon treatment with the cyclotide probes indicated that they are effective in significantly reducing levels of molecular effector cytokines as compared to the myelin oligoglycoprotein-treated cells (Figure 3B). Despite these chemical modifications, the cyclotide probe [T20K,W23Bpa]kB1 exhibited unaltered structural features and retained biological activity and hence it is considered as valid probe for subsequent application in target elucidation.

### Photo-affinity pull down using [T20K]kB1 to identify target protein(s) of cyclotides

**Figure 4:**
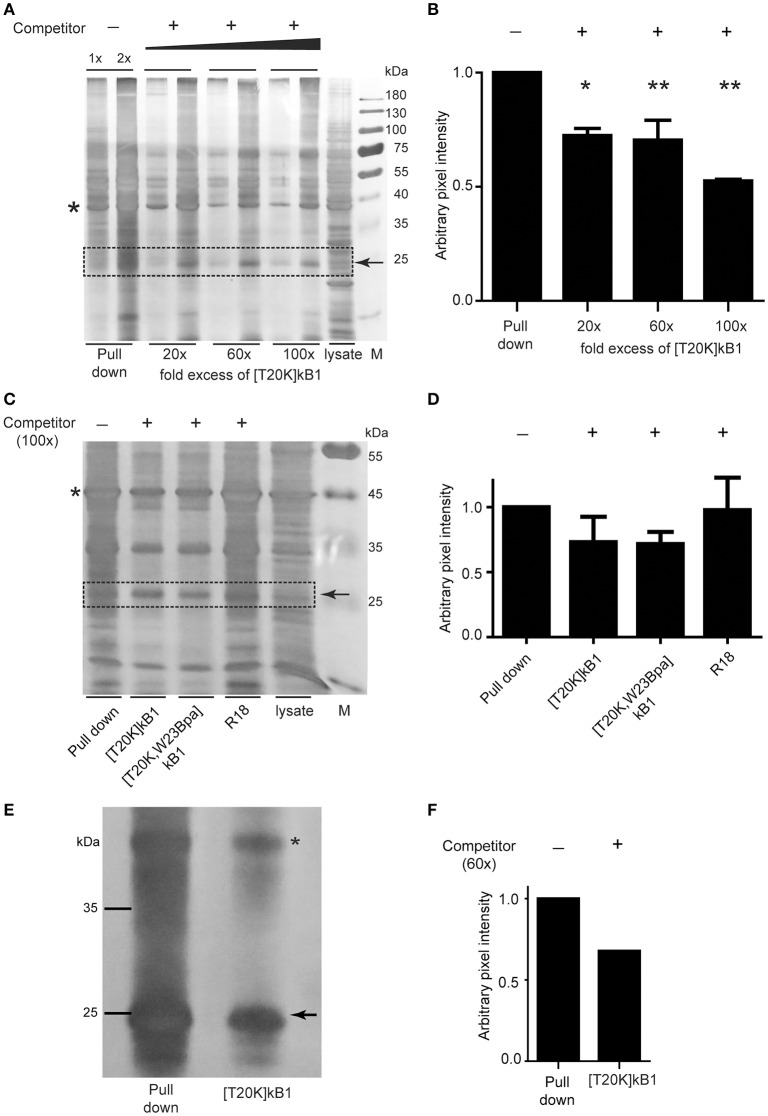
Photo-affinity capture of binding proteins with cyclotide probes and competitor titration for target identification. **(A)** Representative silver-stained gel image of Jurkat cell lysates probed with photo-affinity crosslinker [T20K,W23Bpa]kalata B1 (kB1) and pull down via biotin-linker in the absence (−) and presence (+) of bait competitor [T20K]kB1 at 20-, 60-, and 100-fold molar excess relative to the amount of [T20K,W23Bpa]kB1. **(B)** Exemplarily, a 25 kDa protein band that was affected in intensity by application of competitor which was semi-quantified relative to the pull down sample via pixel analysis of replicated silver gels using ImageJ. Values were normalized to a random internal loading control indicated with an asterisk. **(C)** Total soluble protein lysate isolated from MOG immunized mouse splenocytes (EAE disease inductions) were incubated with photo-affinity probe [T20K,W23Bpa]kB1, crosslinked and pulled down, with or without addition of various competitors [T20K]kB1, [T20K,W23Bpa]kB1 and pan-14-3-3 peptide inhibitor R18 (all at 100-fold molar excess). A representative silver stained protein gel is illustrated. **(D)** Semi-quantitative evaluation of the 25 kDa protein band via pixel analysis as described in **(B)**. **(E)** Photo-affinity pull down experiment on living Jurkat cells in cell culture is illustrated via a silver stained protein gel. After 2 h of incubation with bait peptide and with or without the presence of 60-fold excess of competitor [T20K]kB1, the crosslinker was activated by UV-irradiation. Values were normalized to a random internal loading control indicated with an asterisk. **(F)** Semi-quantitative evaluation of the 25 kDa protein band via pixel analysis as described in **(B)**.

Since commonly observed nonspecific interaction(s) of Bpa probes may bias a direct read-out from whole proteome pull downs, we performed gel-based competition experiments to reduce bias. Competitive validation enabled the study of specific binding proteins. Competitor peptide [T20K]kB1 titrated in at 20-, 60-, and 100-fold molar excess of biotinylated probe [T20K,W23Bpa]kB1, revealed an effect on the band intensity of a 25 kDa protein (Figure 4A). Despite the low resolution of the 1D polyacrylamide gel yielding multiple overlaid proteins in the 25 kDa range, the competition variant enabled interpretation of the protein intensities of the gel bands via semi-quantitative analysis (Figure 4B). Using in-gel trypsin digestion and bottom-up proteomics analysis of the proteins in the gel samples, the family of 14-3-3 proteins (in detail the α/β, ε, θ/τ, γ, η, and ζ/δ out of a total of seven isoforms) were detected in this study besides other prominent proteins such as, RS4X, CAPZB, RL10A, PCBP1, PCBP2, and DCXR (all identifications are reported in Supplementary Data S1). To confirm the identified binding proteins of [T20K]kB1 we performed pull down experiments with an immune proteome isolated from murine experimental autoimmune encephalomyelitis-induced or 2D2-derived splenocytes. Moreover, we explored the presumed binding of 14-3-3 proteins to the probe by specifically modulating 14-3-3 proteins with R18, a peptidic pan-14-3-3 inhibitor, along with competition of cyclotides [T20K]kB1 and [T20K,W23Bpa]kB1. Silver stained protein gels showed that R18 did not alter the intensity of protein bands throughout the sample (Figures 4C,D). The use of splenocyte lysates impeded gel-based protein band quantification by several overlaid proteins in the 25 kDa range. However, in-gel digestion and proteomics analysis of proteins in the corresponding gel band clearly revealed a competition effect in the order [T20K]kB1 < [T20K,W23Bpa]kB1 < R18 for 14-3-3 proteins (Figure 4D and Supplementary Data S2). This finding reflects that the members of the 14-3-3 family bind to cyclotide [T20K]kB1 and its photo-crosslinking derivatives, as well as that they are sensitive to competition with native cyclotides. Moreover, the labeling procedure works reliably with both protein extracts and on living cells in culture. To show that 14-3-3 proteins were labeled and that their retention on the affinity beads is competitively reduced by the presence of a proper excess of competitor, Jurkat cells were incubated with the bait [T20K,W23Bpa]kB1 peptide and with competitor peptide [T20K]kB1 (Figures 4E,F and Supplementary Data S3). Since artifacts may arise from labeling in protein extracts rather than in the more troublesome cell culture samples the obtained results indicate that using protein extracts in photo-affinity labeling experiments is in the presented case a qualified methodology to investigate protein-peptide interactions (Volke et al., 2015).

### Co-precipitation assay for the validation of the cyclotide-target interaction

**Figure 5:**
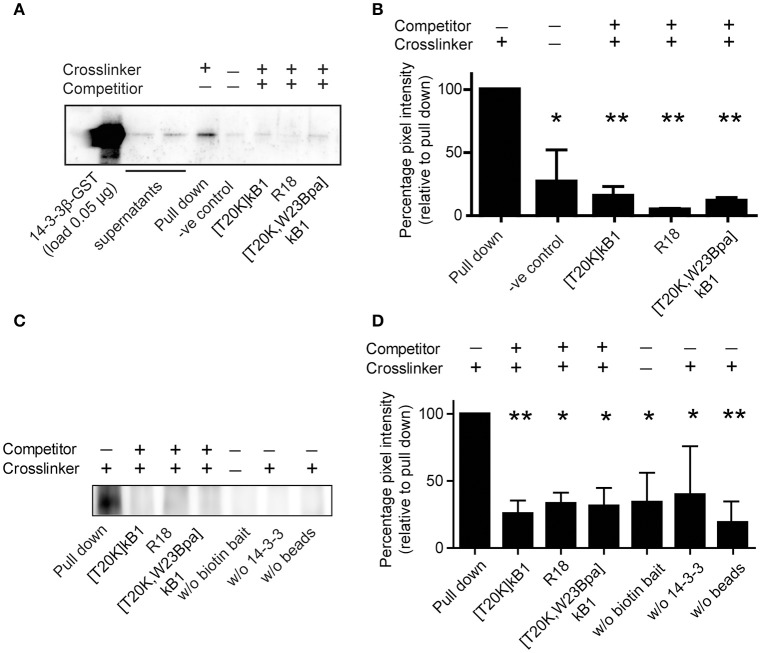
Co-precipitation of purified GST-tagged isoform 14-3-3β with cyclotide probe [T20K,W23Bpa]kB1. **(A)** Protein/peptide interaction was confirmed by co-precipitation of representative recombinant GST-tagged 14-3-3β isoform with biotinylated crosslinker probe [T20K,W23Bpa]kB1. Validation of the interaction was obtained by competition experiments using non-biotinylated competitors [T20K]kB1, R18, and [T20K,W23Bpa]kB1, respectively. Beads and crosslinker probe [T20K,W23Bpa]kB1 only, were used as a control. After precipitation of biotinylated analytes with streptavidin beads, 14-3-3 positive samples were identified using an anti-GST primary antibody. A representative immunoblot is shown. **(B)** Pixel quantification of two independent immunoblots revealed significant reduction of the GST-tagged 14-3-3β isoform in the presence of non-biotinylated competitors. **(C)** *In vitro* co-precipitation was performed using the biotin tag for the precipitation to test for any binding of the probe to the GST tag of the 14-3-3β protein. As control the following conditions were used: (i) samples without biotinylated crosslinker, (ii) samples without 14-3-3β GST protein and (iii) samples without streptavidin beads. A representative immunoblot of three independent experiments is shown. **(D)** Pixel quantification of protein bands of the 55 kDa band (14-3-3β conjugated to a GST-tag and crosslinked to the biotinylated cyclotide probe [T20K,W23Bpa]kB1). Significance was analyzed by one-way ANOVA analysis with Bonferroni *Post-Hoc* test; ^*^P < 0.05, ^**^P < 0.01.

The examined peptide/protein interaction was validated by biochemical co-precipitation using recombinantly expressed 14-3-3 protein. Sequence analysis of human 14-3-3 isoform suggested that they all comprise conserved ligand binding sites (Tzivion et al., 2001). Hence, we chose the representative 14-3-3β isoform, which was incubated with biotinylated compound [T20K,W23Bpa]kB1 with or without competitors for photo-affinity experiments using either the streptavidin or the GST affinity label of the recombinant protein for the precipitation. The β-isoform was considered as prototypic cytosolic 14-3-3 protein (Uhlen et al., 2015) amongst others reported to have strong tissue-specific expression. As seen by immune blotting (Figures 5A,B), and far Western blotting (Figures 5C,D) the binding of probe [T20K,W23Bpa]kB1 to 14-3-3β was significantly altered by the presence of competitors [T20K,W23Bpa]kB1, [T20K]kB1 and the R18 peptide (Figures 5C,D), confirming the cyclotide [T20K]kB1 can bind to 14-3-3 family proteins.

### Cell-based confirmation of the cyclotide/14-3-3 interaction

**Figure 6:**
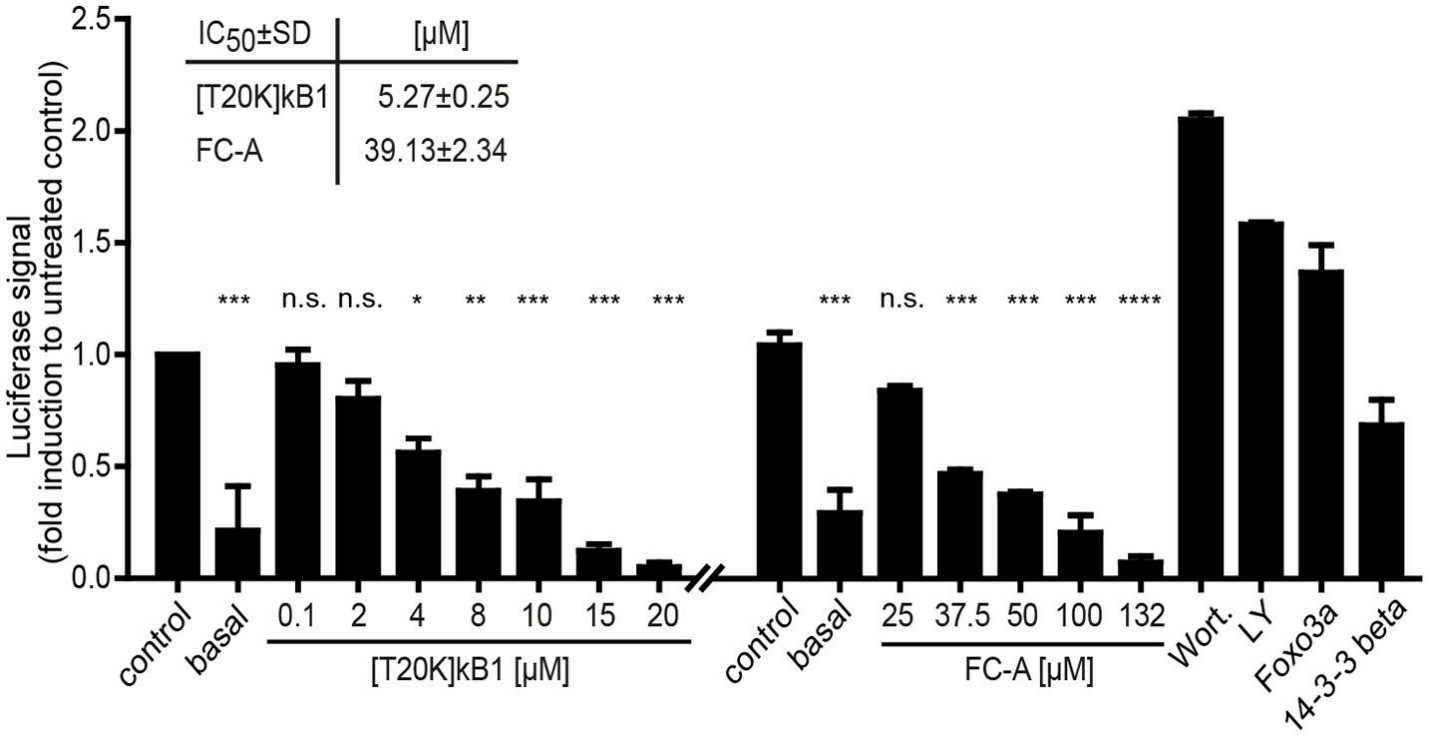
Foxo3a luciferase reporter assay. The modulation of the Akt/PI3K/Foxo3a pathway by the cyclotide [T20K]kB1 binding to 14-3-3 proteins was investigated. HEK293 cells were transiently transfected with Foxo3a-Luc plasmid encoding luciferase under a promotor with Foxo response elements, Foxo3a and 14-3-3β. For control the reporter plasmid (basal value) was also co-transfected with the single functional effector proteins 14-3-3β or Foxo3a. Cells were treated under starving conditions with peptide or fusicoccin-A (FC-A) for 4 h or 24 h, respectively. The 14-3-3 stabilizer compound fusicoccin-A is reported to impact the cellular location of 14-3-3 complexed transcription factors (shown by an independent experiment). Wortmannin (Wort.) and LY294002 (LY) served as positive control compounds inhibiting the phosphoinositol 3-kinase and thus interfering with the nuclear export of Foxo3a. IC_50_-values were calculated of two independent experiments using nonlinear regression with curve fitting and constrains to the top and bottom values. Statistics show one-way ANOVA analysis with Bonferroni *Post-Hoc* test; ^*^P < 0.05, ^**^P < 0.01, ^***^P < 0.001; ^****^P < 0.0001, n.s., not significant.

Due to the lack of appropriate cellular models to study 14-3-3 efficacy in terms of immunosuppression and the fact that the seven protein isoforms are capable to compensate any single 14-3-3 isoform knock-out, a validation assay was conducted using peptide probes and appropriate pathway modulators (positive controls) to study [T20K]kB1-induced 14-3-3 modulation of apoptosis and cell proliferation. 14-3-3 proteins are known to control proliferation and quiescence of immune cells via the Akt/phosphoinositide 3-kinase (PI3K)/Forkhead box protein 3a (Foxo3a) pathway (Oh et al., 2012). Therefore, we utilized a luciferase-based reporter assay to determine whether the cyclotide [T20K]kB1 can modulate Foxo3a cell function on the level of cell proliferation. HEK293 cells were transiently transfected with plasmids encoding luciferase under the control of a Foxo3a promoter, and with 14-3-3β. [T20K]kB1 reduced the transcriptional activity of Foxo3a in a concentration-dependent manner with an IC_50_ of 5.3 ± 0.2 μM (Figure 6), confirming the interaction of cyclotide and 14-3-3 protein in a cellular context. As control compound, the known 14-3-3 modulator fusicoccin-A was also able to reduce the luciferase signal with an IC_50_ of 39.1 ± 2.3 μM, whereas the PI3K inhibitors wortmannin and LY294002 led to increased transcriptional activity by inhibiting the kinase cascade and hence reducing cytosolic p-Foxo3a levels.

## Discussion: application and effectiveness of the method

This study was designed to develop a robust workflow for target elucidation of the bioactive cyclotide [T20K]kB1, a promising preclinical drug candidate for the treatment of autoimmune disorders. Cyclotides comprise one of the largest family of naturally-occurring circular and cystine-knot peptides (Gruber et al., 2008; Hellinger et al., 2015b) and they are therefore considered as representatives for the large group of ribosomally synthesized and post-translationally modified peptides (RiPPs) (Arnison et al., 2013). RiPPs in general and in particular, cyclic or stabilized peptides are considered as highly promising candidates in drug development (Craik et al., 2013; Kaspar and Reichert, 2013). Such bioactive peptides are usually discovered via phenotypic screening of nature-derived compound libraries, a technique which made substantial contributions to drug discovery and development (Bottcher et al., 2010; Nicolaou, 2014; Mullard, 2015). Although the large family of circular and structurally constrained peptides exhibit physico-chemical properties that are *per se* promising and well-suited for modern peptide drug development, little attention has been paid so far to establish methodologies and tools to guide drug development of these peptides (Terstappen et al., 2007; Sletten and Bertozzi, 2009; Rask-Andersen et al., 2011). Macrocyclic and stabilized peptides have reduced amenability to chemical modifications, which renders the design of functional probes for a chemical proteomics strategy a difficult endeavor. For example, cyclic or constrained peptides do not possess an accessible N-terminal site (e.g., cyclotides and orbitides) or they have multiple reactive sites (e.g., defensins, many venom toxins), which limits site-specific labeling via amine-reactive reagents. In addition, common stabilizing scaffolds of nature-derived peptides, such as, the cystine-knot motif of cyclotides and knottins, or common post-translational modifications of RiPPs (e.g., amidation, N-methylation, lactone bridge) impact their structure, conformation and bioactivity, and they impede their chemical synthesis. To overcome these limitations, we used the prototypic cyclotide kalata B1 as a template for the design of photo-affinity labeling probes. A photo-reactive moiety and a lysine residue were incorporated into the cyclic peptide backbone without perturbing the native fold or the overall structural arrangement. One of our probes [(T20K,W23Bpa)kB1] was subject to target deconvolution experiments using cellular protein lysates and cultured cells to characterize and identify its molecular interaction partner. Using a chemical proteomics strategy including synthesis and validation of chemical probes we exemplarily identified the family of 14-3-3 proteins as a possible target of kalata-type cyclotides. Exemplarily, the identified 14-3-3 proteins were considered as representative binding partners for the cyclotide. The generic protein-peptide interaction was validated via molecule competition experiments, by *in vitro* precipitation and at a functional level studying the modulation of signaling associated to apoptosis and the control of cell proliferation.

14-3-3s are ubiquitous adaptor proteins that are involved in cell signaling, cell cycle progression and apoptosis as well as protein-protein interaction and cellular transport (Aghazadeh and Papadopoulos, 2016). The 14-3-3 family is often described as a chimera, since knockout of single isoforms may remain without or with minor phenotypic changes due to functional rescue through the recruitment of other family members. It has been suggested that the seven 14-3-3 isoforms expressed in humans play a pivotal function in cell physiology. Many tumor suppressors or oncogenes are regulated by 14-3-3 proteins, and therefore they are intensively studied as possible agents for cancer therapy and diagnosis (Phan et al., 2015; Aghazadeh and Papadopoulos, 2016). For example, 14-3-3s are also known to be involved in the regulation of cell proliferation and quiescent states in immune cell homeostasis (Oh et al., 2012; Aghazadeh and Papadopoulos, 2016). Our data indicate a biochemical interaction of 14-3-3 proteins and the immunomodulatory cyclotide [T20K]kB1. This immune system modulatory peptide is reported to reduce the symptoms of the EAE disease in mice but there is not much known about the underlying mechanism except that regulatory cytokines such as, IL-2 are significantly downregulated in T-lymphocytes in [T20K]kB1 treated animals.

There are generally two types of 14-3-3 ligands: (i) inhibitors or (ii) stabilizers. 14-3-3 inhibitors such as, the peptides R18 and difopein increase Foxo3a nuclear import to induce pro-apoptotic transcriptional activity (Arrendale et al., 2012). In contrast, 14-3-3 stabilizers enforce a protein-protein interaction and they may account for an increased retention of 14-3-3 complexed proteins in the cytosol or other cellular compartments (Ottmann, 2013; Bier et al., 2015). For example, fusicoccin-A has the capability to enforce protein complex formation of 14-3-3s with transcription factors and to modulate cellular signaling (Skwarczynska et al., 2013; Bier et al., 2015; Milroy et al., 2015). Our results indicate that cyclotides modulate 14-3-3 function in a similar fashion as observed for the 14-3-3 stabilizer fusicoccin-A. In a luciferase-based reporter assay the peptide decreased the transcriptional activity of Foxo3a, which suggests that [T20K]kB1 exerts a stabilizing effect on the 14-3-3/Foxo3a protein complex. Recently, two secondary binding pockets of 14-3-3 proteins were reported which indicate that this protein family is capable of multiple different interactions with small molecules and peptides to modulate protein-protein interactions (Hartman and Hirsch, 2017; Sijbesma et al., 2017). The described biological effect of [T20K]kB1 to interfere with Forkhead box protein dependent transcriptional activity is too limited to provide a basis for interpretation of [T20K]B1 effects on a multifaceted immune environment. Nevertheless, it successfully served for validation of the methodology for target discovery of this cyclotide.

Incidentally, stabilization of protein-protein interaction is a well-known mechanism of other immunosuppressive drugs on the market: cyclosporine A—a nature-derived undecapeptide that historically revolutionized the field of immunopharmacology (Thell et al., 2016)—stabilizes the calcineurin/immunophilin complex. Similarly, rapamycin binds to the protein FK-binding protein 12 which upon acts as an inhibitor for the mTORC complex thus impedes the protein complex cellular function. Interestingly, mizoribine—an approved immunosuppressant small molecule drug—reportedly modulate 14-3-3 protein function, but the role of this interaction for the suppression of cell proliferation by this compound has not been fully explored, yet (Kawasaki, 2009). Cyclotides are anti-proliferative and T-cell modulating compounds, which are described for the treatment of autoimmune disorders especially multiple sclerosis (Thell et al., 2016). On a molecular level they mainly affect the transcription and production of the signature cytokine interleukin-2 of T-lymphocytes. Since interleukin-2 transcription can be regulated via a signaling cascade of Akt/PI3K through 14-3-3/Foxo3a and IκB it is conceivable that the interaction of the cyclotide [T20K]kB1 with 14-3-3 proteins mediates the described effects (Oh et al., 2012). This observation may serve as starting point to investigate in future studies whether cyclotides can modulate 14-3-3 proteins *in vivo* and whether this interaction contributes to the promising disease suppression of multiple sclerosis in the experimental autoimmune encephalomyelitis mouse model.

This study was focused on target identification in an immune cell related biological context and used specially designed and bioactive photo-affinity probes as a tool for target elucidation. The reported 14-3-3 proteins are one example of a protein interaction partner of a native cyclotide, but work from our own laboratory suggests that cyclotides can also bind to and modulate the function of enzymes such as, proteases (Hellinger et al., 2015a) and G protein-coupled receptors (Koehbach et al., 2013; Fahradpour et al., 2017). The presented workflow has limitations to detect membrane-associated proteins or proteins with heavy post-translational modifications. A more suitable approach to identify further protein targets of cyclotides would be quantitative mass spectrometry of protein pull downs using affinity or photo-affinity probes. Furthermore, to identify possible cyclotide-transmembrane protein interactions the presented methodology would require further optimization.

Meanwhile, plant-derived cyclotides are recognized for their pharmaceutical potential. The drug candidate [T20K]kB1 halted the progression of typical symptoms in a mouse model of multiple sclerosis after oral administration (Thell et al., 2016). Additionally, cyclotides are considered as promising templates for the design of stabilized, orally active peptide epitopes (Jagadish and Camarero, 2010; Craik et al., 2012). The cystine-knot motif is capable of tolerance to the incorporation of several amino acid long sequences without impacting its endogenous structure and stability. Using this so-called “grafting approach” several peptide probes based on the cyclotide scaffold have been designed to target G-protein-coupled receptors. For instance, agonists and antagonists for the endothelial growth factor-A, the CXC-chemokine receptor 4, the melanocortin receptor 4 and the bradykinin 1b receptor were recently reported (Aboye et al., 2012; Eliasen et al., 2012; Wong et al., 2012; Getz et al., 2013).

At a more general level, circular and stabilized peptides and RiPPs are promising candidates to have significant impact on future drug development efforts. Their unique topology and inherent stability may complement the existing set of bioactive (small) molecules used in the screening of drug candidates. Bioactive RiPP discovery is often inspired by phenotypic screening of nature-derived peptide libraries from plant, fungal or animal origin. For instance this led to the development of peptide pharmaceuticals such as, Linzess®, Prialt®, and SandImmune® (Craik et al., 2012, 2013; Arnison et al., 2013; Vizcaino et al., 2013; Thell et al., 2016). However, at the stage of drug discovery it is imperative to ensure further development by the identification of drug targets for such molecules. In this study, we developed respective tools and a methodology to link a peptide drug candidate derived from a phenotypic drug screen to a molecular target. Although target elucidation is still under prioritized in pre-clinical drug development, it is still considered as the “holy grail” in drug discovery and hence the presented approach will hopefully guide future development of promising circular and cystine-stabilized peptide drug candidates.

## Concluding remarks: advantages and limitations of the method

Many current peptide drugs in clinical use were inspired from naturally-occurring compounds and it is to be expected that the trend to explore natural bioactive libraries as a source for novel drug candidates is increasing, which will further stimulate progress of circular and stabilized peptides in drug development. Overall, the presented workflow comprises the design and preparation of circular and stabilized peptide probes and their validation for the purpose of target identification. For a prototypic cyclotide we provided a procedure to validate an identified target protein to enable further studies on the drugs mechanism. We believe this work can be used as guidance for target deconvolution of other bioactive RiPPs and in particular for cyclotides identified via phenotypic drug screens. The main limitations of this approach are associated with the probe design. It is imperative to experimentally validate the amenability of the chemical probes to ensure their structural integrity and retained biological activity. Hopefully, with increased availability of structure-activity relationship data of cyclotides and other RiPPs, it will be possible to narrow down possible sites for modification based on sequence/structure comparison.

## Author contributions

RH performed peptide synthesis, folding and purification of peptides and photo-crosslinker probes; he also performed target identification and proteomics analysis. KT evaluated the bioactivity of peptide probes and implemented experiments with splenic immune cells. MV assisted characterization of peptides. SG analyzed peptide structures by NMR and performed data evaluation. DK provided recombinant protein and assisted interaction studies. CB designed the peptide synthesis and provided the thioester resin. UG and TM, assisted peptide synthesis. CG, was responsible for research design, project funding, manuscript writing, and the overall coordination. All authors reviewed the results and approved the final version of the manuscript.

### Conflict of interest statement

CG is scientific advisor of Cyxone AB. The other authors declare that the research was conducted in the absence of any commercial or financial relationships that could be construed as a potential conflict of interest.

## Abbreviations

RiPPs: ribosomally synthesized and post-translationally modified peptides
kB1: kalata B1
Bpa: para-benzophenylalanine.
